# The N-glycosylation of Equine Tetherin Affects Antiviral Activity by Regulating Its Subcellular Localization

**DOI:** 10.3390/v12020220

**Published:** 2020-02-16

**Authors:** Bowen Bai, Xue-Feng Wang, Mengmeng Zhang, Lei Na, Xiangmin Zhang, Haili Zhang, Zhibiao Yang, Xiaojun Wang

**Affiliations:** 1 State Key Laboratory of Veterinary Biotechnology, Harbin Veterinary Research Institute of the Chinese Academy of Agricultural Sciences, Harbin 150069, China; byrne0513@gmail.com (B.B.); wangxuefeng@caas.cn (X.-F.W.); zmm973898307@163.com (M.Z.); nalei@caas.cn (L.N.); 13849116520@163.com (X.Z.); zhanghaili@caas.cn (H.Z.); 2 Shanghai Key Laboratory of Veterinary Biotechnology, School of Agriculture and Biology, Shanghai Jiao Tong University, Shanghai 200240, China; zbyang@sjtu.edu.cn

**Keywords:** tetherin, EIAV, N-glycosylation, antiviral function, traffic

## Abstract

Tetherin is an interferon-inducible type II transmembrane glycoprotein which inhibits the release of viruses, including retroviruses, through a “physical tethering” model. However, the role that the glycosylation of tetherin plays in its antiviral activity remains controversial. In this study, we found that mutation of N-glycosylation sites resulted in an attenuation of the antiviral activity of equine tetherin (eqTHN), as well as a reduction in the expression of eqTHN at the plasma membrane (PM). In addition, eqTHN N-glycosylation mutants colocalize obviously with ER, CD63, LAMP1 and endosomes, while WT eqTHN do not. Furthermore, we also found that N-glycosylation impacts the transport of eqTHN in the cell not by affecting the endocytosis, but rather by influencing the anterograde trafficking of the protein. These results suggest that the N-glycosylation of eqTHN is important for the antiviral activity of the protein through regulating its normal subcellular localization. This finding will enhance our understanding of the function of this important restriction factor.

## 1. Introduction

Tetherin (also known as CD317, BST-2 or HM1.24) is a type II transmembrane protein induced by interferon. It is a heavily glycosylated integral membrane protein, and has an unusual topology [1], consisting of a short N-terminal cytoplasmic tail (CT) linked to an alpha-helical transmembrane anchor (TM), an extracellular domain with two potential N-glycosylation sites, a predicted coiled-coil and finally a C-terminal glycophosphatidyl-inositol (GPI)-linked lipid anchor that is believed to ensure incorporation of tetherin into cholesterol-rich lipid rafts [2].

Tetherin has been found to inhibit human immunodeficiency virus-1 (HIV-1) particle release in the absence of Vpu [3], and ever more reports demonstrate that tetherin not only restricts the release of lentiviruses, but also has been found to limit the release of retroviruses [4], arenaviruses [5], filamentous viruses [6] and influenza viruses through tethering them to the cell membrane with its unusual topology [7]. This broad-spectrum inhibition of virus particle release by tetherin via the “physical tethering” model indicates that this restriction is independent of specific interactions with viral proteins [8]. Many viruses have evolved countermeasures against tetherin by encoding antagonists. For example, both HIV-1 Vpu and HIV-2 Env are known to influence tetherin transport to the plasma membrane (PM) and to retain it in a perinucleolar compartment, and Vpu, but not Env, also promotes the degradation of tetherin, suggesting that it uses more than one mechanism to counteract restriction from this protein [9].

Tetherin has been identified from diverse species, including horses, cats, non-human primates and rodents [10]. The function of most tetherins of different origins share antiviral function, although their sequence differences are large. Tetherin passes the trans-Golgi network (TGN), the early and recycling endosomes, and cycles between these cytoplasm compartments and the PM by endocytosis, finally localizing to the PM, where it is able to exert its antiviral function [11].

One of the most common posttranslational modifications, N-glycosylation, which occurs in the endoplasmic reticulum (ER) and the Golgi apparatus during protein synthesis and is important for the stability of numerous glycoproteins [12]. Kupzig et al. found that N-glycosylation of tetherin ensures its normal expression and is required for the entry of tetherin into the secretory pathway [13]. Mature human and feline tetherins harbor N-glycosylation at conserved asparagine residues in their extracellular domains [14,15]. Some previous studies report that N-glycosylation is required for the correct transport and folding of the human tetherin molecule, and may play a specific role in tetherin antiviral activity [8,16]. However, other studies did not find any effect on virus restriction following the mutation of the tetherin N-glycosylation sites [17,18,19]. Accordingly, whether or not N-glycosylation plays a crucial role in the antiviral activity of tetherin, and what its detailed functional contribution is remain unclear.

In this study, we confirm that equine tetherin (eqTHN) has two N-glycosylation sites at 51 and 78. Furthermore, our results show that substitution mutation of eqTHN at N-glycosylation sites changes the subcellular localization of the protein and causes it to lose its antiviral function. This loss of restriction activity of eqTHN following loss of N-glycosylation may be as a result of the protein being unable to reach the PM, where it would normally carry out its antiviral function.

## 2. Materials and Methods

### 2.1. Plasmids

The equine tetherin gene described previously was cloned into a pcDNA3.1-FLAG vector via the *Not*I and *Xho*I restriction sites to construct the expressing plasmids pcDNA3.1-eqTHN-FLAG encoding a FLAG tag inserted between residues 4 and 27 as described previously [20]. The plasmid was used as a sample to generate the asparagine (N) mutant constructs pcDNA3.1-eqTHNN51A-FLAG, pcDNA3.1-eqTHNN78A-FLAG and pcDNA3.1-eqTHNN51/78A-FLAG via two step polymerase chain reaction (PCR) using a KOD FX system (Toyobo, Osaka, Japan). These plasmids were used to perform western blotting, RT and confocal. The primers generated for these mutant constructs are as follows. For eqTHN N51A: forward, 5′-GTCGCGCCGGCACCCACTTTCTGGA-3′, reverse, 5′-TGGGTGCCGGCGCGACACTCCTGCTC-3′; for eqTHN N78A: forward, 5′-TCTGCGCCCAGACTGTGGTGACCCT-3′, reverse, 5′-CAGTCTGGGCGCAGATGGCAGCCT-3′. The equine tetherin gene was cloned into a pcDNA3.1-FLAG vector to construct the expressing plasmids pcDNA3.1-eqTHNe-FLAG encoding a FLAG tag inserted between residues 243 and 267. This plasmid was used as a sample to generate the asparagine (N) mutant constructs pcDNA3.1-eqTHNeN51A-FLAG, pcDNA3.1-eqTHNeN78A-FLAG and pcDNA3.1-eqTHNeN51/78A-FLAG. These plasmids were used to perform flow cytometry. The primers generated for pcDNA3.1-eqTHNe-FLAG are as follows, forward, 5′-GTCGCGCCGGCACCCACTTTCTGGA-3′, reverse, 5′-GTCGCGCCGGCACCCACTTTCTGGA-3′. The same primers previously described for generating pcDNA3.1-eqTHN-FLAG mutants were used to generate for pcDNA3.1-eqTHNe-FLAG mutants. The constructed plasmids were transformed into *E. coli* DH5α cells (TaKaRa, Dalian, China) for amplification and all positive clones identified by PCR, and positive clones were sent to Tsingke (Harbin, China) for sequencing. Codon-optimized equine infectious anemia virus (EIAV) Gag was described in previous study [21].

### 2.2. Cell Culture and Transfection

Human embryonic kidney 293T (HEK293T) cells were cultured in Dulbecco’s modified Eagle’s medium (GIBCO, Thermo Fisher Scientific, Waltham, MA, USA) containing 10% Fetal Bovine Serum (FBS) and 1% penicillin-streptomycin. The HEK293T cells were transiently transfected with the indicated plasmids using PolyJet in vitro DNA transfection reagent (SignaGen, Rockville, MD, USA).

### 2.3. Western Blotting

At 48 h posttransfection, the culture supernatants were collected and the cells were lysed in buffer containing 150 mM Tris-HCl (pH 7.6), 50mM NaCl, 5mM ethylene diamine tetraacetic acid (EDTA), and 1% Triton X-100. The culture supernatant was centrifuged at 12,000× *g* for 5 min at 4 °C to remove cell debris and centrifuged at 20,000× *g* for 2 h at 4 °C again to precipitate. The proteins in the supernatant precipitates and cell lysates were separated by SDS-S-polyacrylamide gel electrophoresis (PAGE), transferred to polyvinylidene difluoride (PVDF) membranes (Millipore, Darmstadt, Germany), and blocked with 3% bovine serum albumin (BSA) in phosphate-buffered saline (PBS) for 2 h. And then membranes were incubated for 2 h with the appropriate primary antibodies. All tetherin proteins with FLAG tags were detected using a mouse monoclonal anti-FLAG antibody (Sigma, St. Louis, MO, USA), followed by a secondary goat anti-mouse IRD800-conjugated monoclonal antibody (Sigma). The anti-actin polyclonal antibody was obtained from Sigma. Gag proteins were detected using a mouse anti-p26 monoclonal antibody (9H8), followed by a secondary goat anti-mouse IRD800-conjugated antibody (Sigma). All experiments were performed at least in triplicate.

### 2.4. Flow Cytometry

HEK293T cells were transfected with various eqTHN-expressing plasmids with FLAG peptide in extracellular domain for 24 h. After fixation with 4% paraformaldehyde, the cells were incubated with the mouse anti-FLAG antibody (Sigma) at 1:1000 dilution for 1 h. After washing, the cells were incubated with Alexa Fluor 488 goat anti-mouse IgG (H + L) secondary antibody (Invitrogen, Waltham, MA, USA) at 1:1000 dilution for 1 h. The mean fluorescence intensity of eqTHN localization on the cell surface was then determined by flow cytometry.

### 2.5. Immunofluorescence Assay

HEK293T cells grown on polystyrene coverslips (NEST Biotechnology, Wuxi, China) were transfected with the expression plasmids using PolyJet in vitro DNA transfection reagent (SignaGen, Rockville, MD, USA), following the manufacturer’s protocol. Cells were washed with phosphate-buffered saline (PBS) at 48 h posttransfection, followed by fixing in 4% (vol/vol) formaldehyde (Beyotime, Nanjing, China) for 15 min at room temperature. The cells were blocked with 3% (wt/vol) BSA in PBS for 2 h. The cells were subsequently immunolabeled with primary antibodies for 1 h at room temperature at the following dilutions: the mouseanti-FLAG antibody (Sigma, St. Louis, MO, USA), 1:1000, the rabbit anti-KDEL (Abcam, Cambridge, UK), 1:500, the rabbit anti-CD63 (Abcam, Cambridge, UK), 1:500, the rabbit Rab 5a (Proteintech, Wuhan, China), 1:500, the rabbit Rab 7a (Proteintech, Wuhan, China), 1:500, the rabbit LAMP1 (Proteintech, Wuhan, China), 1:500. Cells were then immunolabeled with secondary antibodies for 1h at room temperature at the following dilutions: Alexa Fluor 488 goat anti-mouse IgG (H + L) secondary antibody (Invitrogen, Waltham, MA, USA), 1:1000, goat anti-rabbit Alexa Fluor 568 secondary antibody (Thermo Scientific, Waltham, MA, USA) for 1h. Nuclei were stained with 2-(4-amidinophenyl)-6-indolecarbamidine dihydrochloride (DAPI) (Beyotime, Nanjing, China) for 15 min. Images were captured using a confocal microscope (LSM 880; Zeiss, Jena, Germany). Pearson correlation coefficients for eqTHN and the EIAV Gag or cytoplasmic organoids were analyzed for three suspected colocalization points using ZEN software. Pearson’s correlation coefficient is the covariance of the two variables divided by the product of their standard deviations. The form of the definition involves a “product moment”, that is, the mean (the first moment about the origin) of the product of the mean-adjusted random variables. It is a simple way of measuring the relationship between fluorescence intensities to calculate colocalization [22].

### 2.6. Antibody-Antigen Uptake Assay

HEK293T cells were transfected with pcDNA-eqTHNe-FLAG or its variants, at 24 h posttransfection, cells were incubated with the mouse anti-FLAG antibody (Sigma, St. Louis, MO, USA) at 4 °C for 1 h. After complete removal of the antibody and washing three times with PBS, cells were cultured with DMEM medium at 4 °C or 37 °C for an additional 60 min. Cells were then fixed with buffered 4% formaldehyde (Beyotime, Nanjing, China). Cells were stained with Alexa Fluor 488 goat anti-mouse IgG (H + L) (Invitrogen, Waltham, MA, USA), 1:1000 secondary antibody (Abcam, Cambridge, UK). The eqTHN localization on the cell surface was determined by flow cytometry.

### 2.7. Peptide-N-Glycosidase F (PNGase F) Digestion of eqTHN

HEK293T cells were transfected with either WT or eqTHN mutants, lysed with 1% NP40 in PBS for 5 min. Lysates were incubated in glycoprotein denaturing buffer at 100 °C for 15 min to denature the proteins. The reaction was carried out in a volume of 20 μL, including 2 μL 10× G7 reaction buffer, 2 μL 10% NP40 and 16 μL lysate, and incubated with 1000 U of PNGase F (New England Biolabs, Ipswich, MA, USA) at 37 °C for 2 h and then western blotting analysis was carried out with anti-FLAG antibody.

### 2.8. RT Activity Assay

EIAV Viral reverse transcriptase (RT) was rescued from the cell culture supernatants and quantified using an RT activity kit (Reverse Transcriptase Assay, Colorimetric kit, Roche, Basel, Switzerland) as recommended by the manufacturer. The detection protocol has been described in a previous study [23]. The RT activities are expressed as optical density (OD) values, which were determined based on the absorbance at 405 nm.

### 2.9. Statistical Analysis

Statistical analysis was conducted using GraphPad Prism, version 5 (Graph Pad Software, San Diego, CA, USA) and Microsoft Excel (Microsoft, 2010). Data shown give the averages of three independent experiments, and error bars from three independent experiments. ***, *p* < 0.001; **, *p* < 0.01; *, *p* < 0.05; ns, not significant.

## 3. Results

### 3.1. eqTHN is Modified by N-Glycosylation at Residues N51 and N78

**Figure 1:**
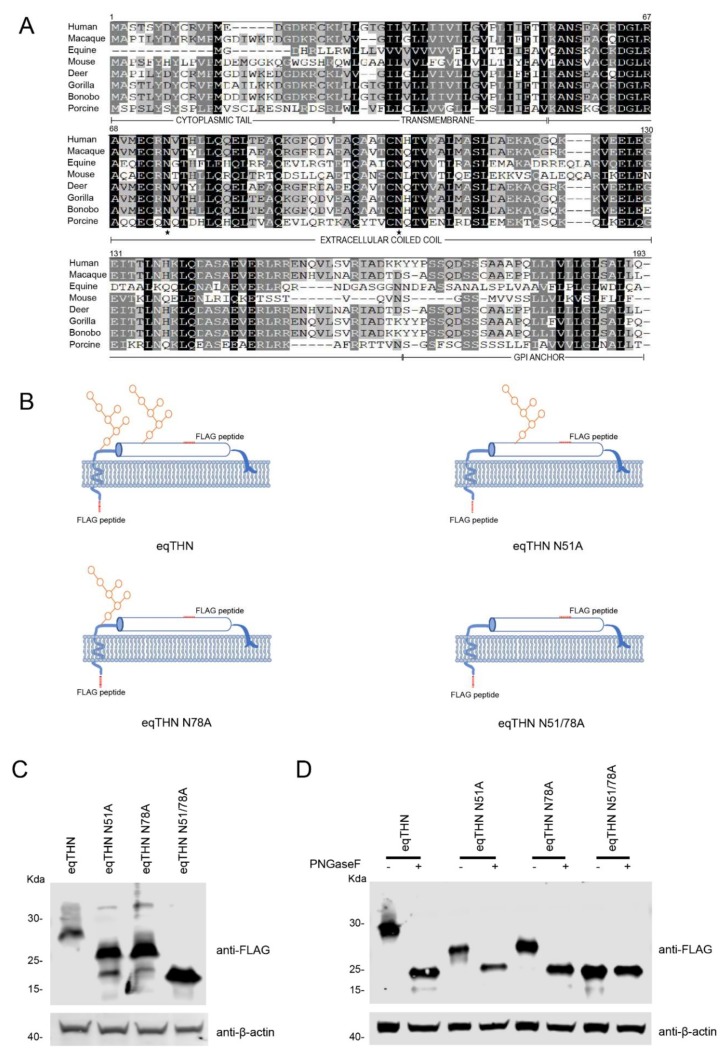
eqTHN is glycosylated at residues N51 and N78. (**A**) Amino acid sequence alignment of human, macaque, equine, mouse, deer, gorilla, bonobo and porcine tetherins using Megalign. The sequences were obtained from the National Center for Biotechnology (NCBI). The consensus asparagine (N) residue lies within the underlined N-glycosylation motif (NxS/T). Identical amino acids are shaded in black, and conserved or similar residues are shown in dark gray. Dashes represent gaps. The transmembrane region, extracellular coiled-coil domain and GPI anchor are marked under the consensus sequences. The N-glycosylation sites in the extracellular coiled-coil domain are indicated by black stars. (**B**) Schematic representation of the eqTHN constructs used in this study. (**C**) The pcDNA3.1-eqTHN-FLAG and N-glycosylation variant plasmids (each 1μg) were transfected into HEK293T cells. At 48 h posttransfection, cells were harvested and then identified using western blotting with an anti-FLAG antibody, with actin as a control. (**D**) HEK293T cells transfected with WT eqTHN and N-glycosylation variants. At 48 h posttransfection, cell lysates were treated with glycosidase F (PNGase F) for 1 h. The reaction products were immunoblotted with anti-FLAG monoclonal antibody. This experiment was repeated three times, and the representative data are shown.

The primary amino acid sequence of tetherins from eight different species are given in Figure 1A. All sequences were obtained from NCBI. To identify potential N-glycosylation sites in the tetherins from different species, we analyzed the amino acid sequences of tetherin using NetNGlyc 1.0 (http://www.cbs.dtu.dk/services/NetNGlyc/). Intriguingly, tetherins from all the eight species investigated possess two potential N-glycosylation sites, separated by 27 amino acids, in the extracellular domains of the proteins. In order to verify the potential N-glycosylation sites of 51NGTH54 and 78NQTV81 in eqTHN, we substituted alanine for asparagine at these sites using site-directed mutagenesis to generate the mutants eqTHN N51A, eqTHN N78A and eqTHN N51/78A (Figure 1B). In HEK293T cells transiently transfected with either WT eqTHN or eqTHN mutants, the results showed that molecular weight of eqTHN mutants were significantly reduced compared with the WT eqTHN (Figure 1C). In a subsequent experiment, the total proteins prepared from HEK293T cells transiently transfected with either WT eqTHN or mutants were treated with PNGase F, which specifically cleaves N-glycosylations including those on complex carbohydrate chains, the molecular weight of WT eqTHN, eqTHN N51A and eqTHN N78A were greatly reduced, while there was no obviously impact on eqTHN N51/78A. Meanwhile, treatment of the WT eqTHN, eqTHN N51A and eqTHN N78A proteins with PNGase F reduced the molecular weight of these proteins to about that of eqTHN N51/78A (Figure 1D). These results suggest that eqTHN is modified with N-glycosylation at the two sites N51 and N78, and that eqTHN has only these two N-glycosylation sites.

### 3.2. N-Glycosylation Affected the Antiviral Function of eqTHN

**Figure 2:**
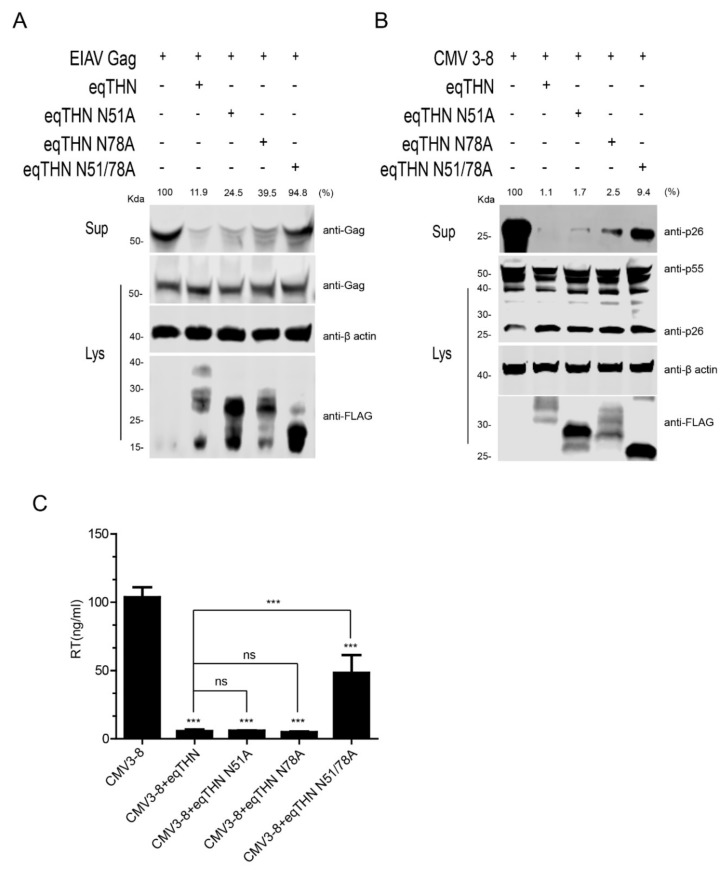
N-glycosylation affects the antiviral activity of eqTHN. (**A**) HEK293T cells were transfected with 1 μg of EIAV gag and 1 μg of expression vector carrying either WT eqTHN or one of its N-glycosylation mutants. At 48 h posttransfection, cultured supernatants were ultracentrifuged to concentrate the released Gag protein. Western blotting, using anti-EIAV p26 monoclonal antibody, was used to detect the presence of Gag proteins in the supernatants and cell lysates. (**B**) HEK293T cells were transfected with 1 μg of infectious EIAV clone (pCMV3-8) and 1 μg of WT eqTHN or one of its mutants. At 48 h posttransfection, cultured supernatants were ultracentrifuged to concentrate the virion particles. Viral CA protein (p26) in the supernatant and Gag protein (p55) in the cell lysates were analyzed by western blotting using anti-EIAV p26 monoclonal antibody. (**C**) Released virions from HEK293T cells transfected with 1 μg of pCMV3-8 and 1 μg of either WT eqTHN or one of its mutants were analyzed by reverse transcriptase (RT) activity at 48 h posttransfection. Virions were analyzed using western blotting to detect viral Gag and CA proteins’ band intensity by Image Studio. Error bars from three independent experiments. *** *p* < 0.001; ns, not significant.

Previous studies have conflicting results regarding the importance of N-glycosylation in tetherin. To investigate the function of N-glycosylation in the antiviral activity of eqTHN, we used an EIAV Gag-based virus-like particle (VLP) release assay to evaluate the restriction ability of eqTHN, which is commonly used in the field [10]. HEK293T cells were co-transfected with EIAV Gag and WT eqTHN or mutant eqTHN expression plasmids. The amounts of Gag in the cell lysate and culture supernatant were measured by western blotting at 48 h posttransfection. As shown in Figure 2A, the overall levels of Gag in the cell lysate were not affected by the N-glycosylation mutants. However, the level of Gag released into the supernatant was reduced in the cells expressing WT eqTHN, eqTHN N51A or eqTHN N78A, but not in cells expressing eqTHN N51A/N78A. This result suggests that the single N-glycosylation mutants (N51 or 78A) had only a small effect on the antiviral activity of eqTHN, while the N51/78A double mutant was almost completely inactive. To generalize these observations to a system used more routinely to produce infectious virions, we transfected the EIAV infection clone pCMV3-8 with either WT eqTHN or its N-glycosylation mutants into HEK293T cells [24]. At 48 h posttransfection, the cell lysate and the virions in the supernatant were collected, and expression of Gag protein (p55) and the viral capsid (p26) cleaved from p55 were assessed using western blotting. The results show that compared with WT eqTHN, eqTHN N51A and eqTHN N78A, the release of virions increased obviously in cells transfected with eqTHN double mutant N51A/N78A, which suggests that double mutation of N-glycosylation can significantly impact the antiviral function of eqTHN (Figure 2B). We confirmed these results by analyzing the reverse transcriptional (RT) activity of virions released into the supernatant (Figure 2C). This technique is often used to detect and quantify retrovirus progeny in cell cultures [25]. Taken together, these results strongly suggest that N-glycosylation of eqTHN influences its antiviral activity.

### 3.3. The N-Glycosylation of eqTHN is Required for Localization to the PM

**Figure 3:**
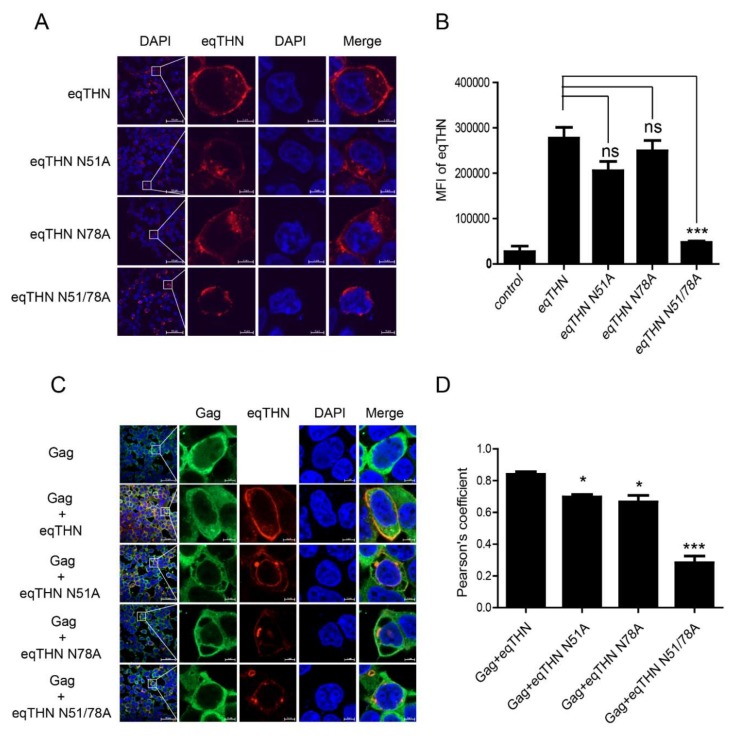
Destruction of N-glycosylation influences localization of eqTHN to the PM. (**A**) HEK293T cells transfected with 1 μg of either WT eqTHN, eqTHN N51A, eqTHN N78A or eqTHN N51/78A were examined using confocal microscopy with surface staining and intracellular staining: blue, cell nucleus; red, eqTHN protein tagged with FLAG. (**B**) HEK293T cells transfected with 1μg of plasmids expressing either pCDNA-eqTHNe-FLAG or its N-glycosylation mutants, and 1μg of plasmid expressing pcDNA3.1-FLAG as a control. At 24 h posttransfection, the cells were harvested and the mean fluorescence intensity of eqTHN on the cell surface was analyzed by flow cytometry using an anti-FLAG antibody. Error bars indicate the SEM from three independent experiments. (**C**) HEK293T cells were transfected with 1 μg of EIAV gag and 1 μg of eqTHN variants, and were examined using confocal microscopy: blue, cell nucleus; red, eqTHN protein tagged with FLAG; green, EIAV Gag. Each experiment was performed three times, and a representative result is shown. (**D**) Pearson correlation coefficient values for the colocalization of Gag and eqTHN at the PM. Error bars from three independent experiments. ***, *p* < 0.001; *, *p* < 0.05; ns, not significant.

Because tetherin needs to be physically present at the PM to restrict the release of virus particles [20], we speculated whether N-glycosylation affects the subcellular localization of eqTHN and in this way influences its antiviral function. In cells expressing WT eqTHN, the tetherin was found to be located mainly at the PM (Figure 3A). Tetherins in cells expressing either eqTHN N51A or N78A appeared partly in the cytoplasm while the double mutant eqTHN N51A/N78A exhibited a puncta-like distribution, and was found mostly in the cytoplasm with an obvious reduction in the amount of protein localized to the PM. The results suggest that N-glycosylation of the two N-glycosylation sites on eqTHN influences the subcellular localization of the protein. In order to further explore the impact of N-glycosylation on localization of eqTHN to the PM, a FACS analysis to evaluate cell surface proteins was carried out. The histograms comparing the levels of surface eqTHN are shown in Figure 3B. Slightly more WT eqTHN is located at the cell surface than either of the N51A and N78A single site mutants. Much less of the double mutant N51/78A localized to the cell surface, suggesting that the localization of eqTHN to the cell surface is significantly influenced by N-glycosylation modification.

Tetherin has been shown to restrict the release of various lentiviruses by inhibiting the release of budding virions from infected cells [26]. To understand how eqTHN blocks EIAV release and to determine whether subcellular localization impacts the ability of the protein to restrict the virus, we investigated whether WT eqTHN or its N-glycosylation mutants could retain EIAV Gag on the cell surface. The colocalization of the WT eqTHN or eqTHN mutants with EIAV Gag also was examined (Figure 3C). When WT eqTHN and Gag proteins were expressed together, most of eqTHN and the EIAV Gag were colocalized at the PM. However, when eqTHN mutants were cotransfected with EIAV Gag, Gag proteins were still observed at the PM, while N-glycosylation site mutants were expressed in the cytoplasm. Quantitative analysis showed obvious colocalization between Gag and WT eqTHN and N51A or N78A mutants at the PM but not the double N51/78A mutants (Figure 3D). These results suggest that the double N-glycosylation mutant eqTHN may lose part of its ability to inhibit the release of Gag, and that this reduction in antiviral activity may be due to the mutations in the eqTHN N-glycosylation sites markedly altering it subcellular localization.

### 3.4. N-Glycosylation Impacts the Normal Trafficking of eqTHN

**Figure 4:**
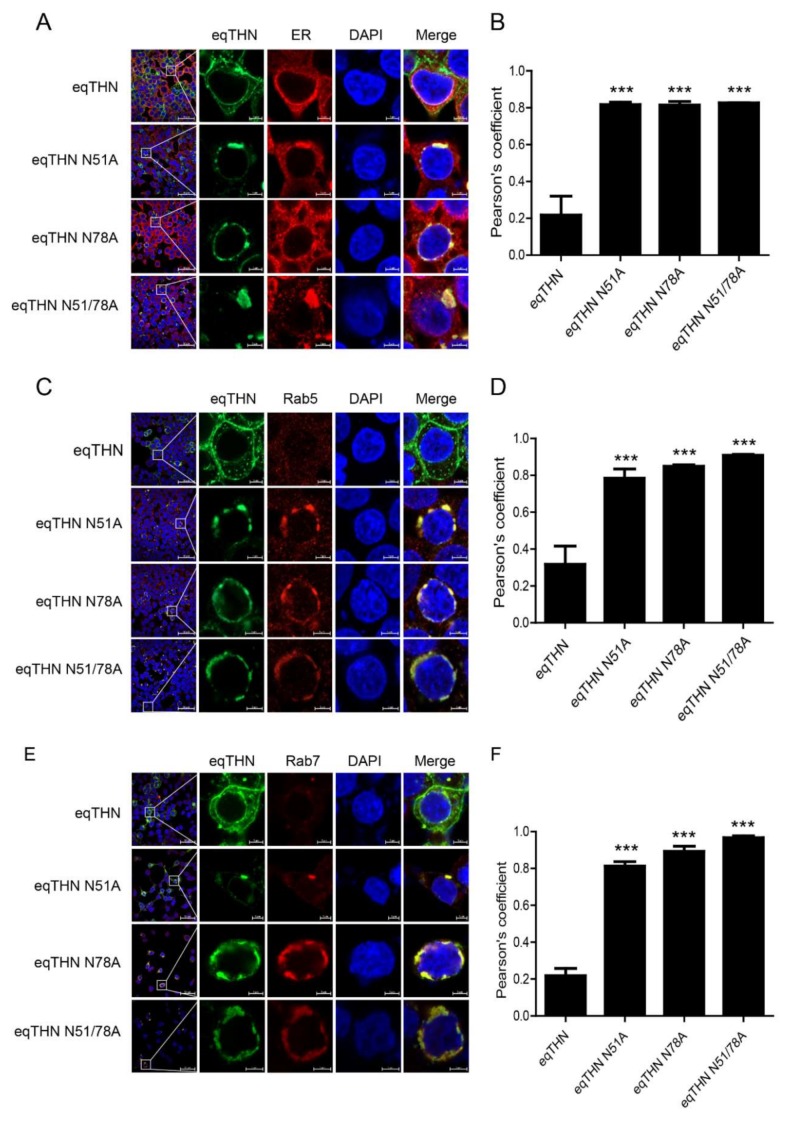

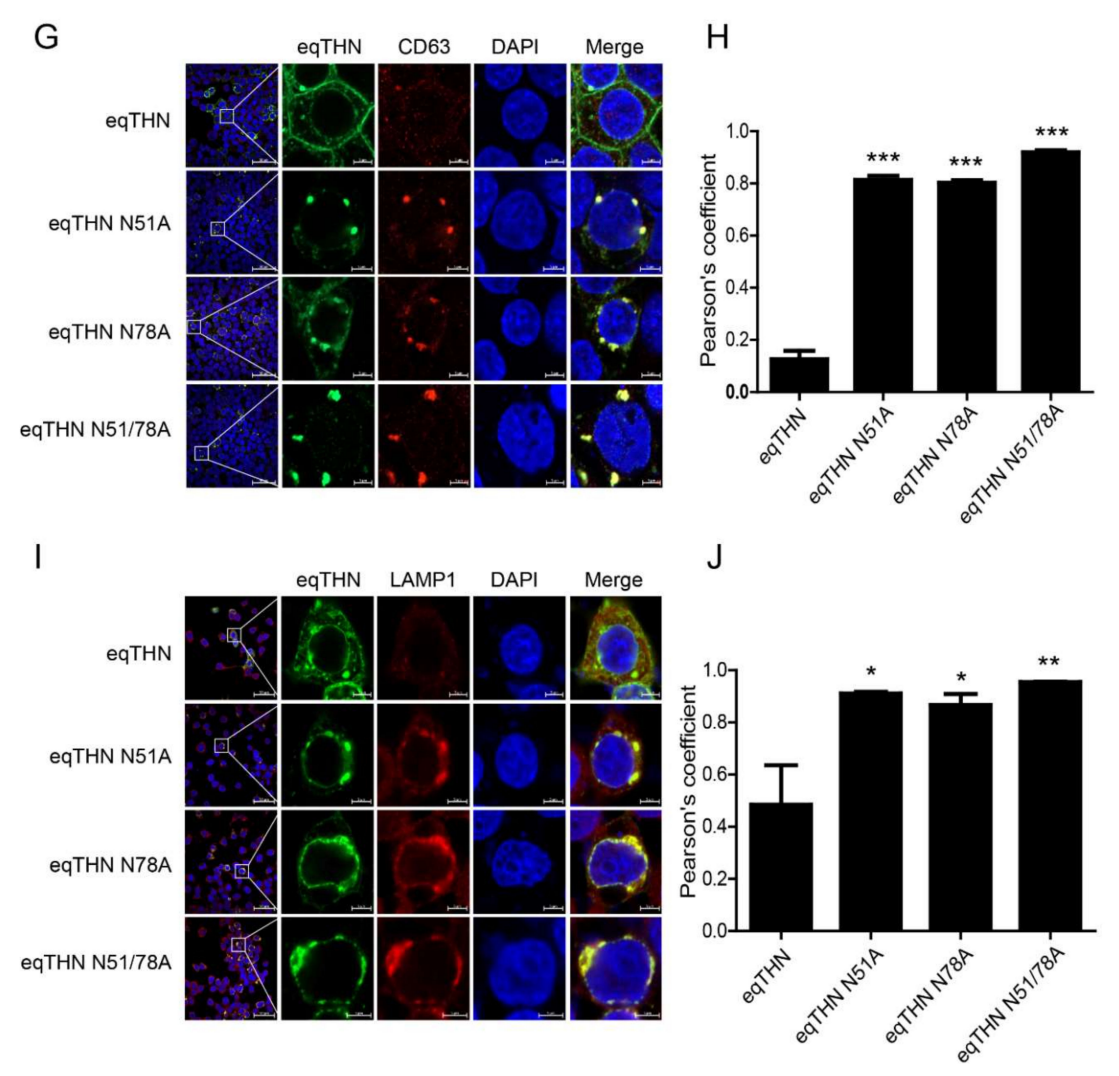
N-glycosylation modification mediates the normal trafficking of eqTHN in cells. HEK293T cells transfected with 1 μg of plasmids expressing eqTHN or its N-glycosylation mutants were investigated using confocal microscopy: blue, cell nucleus; green, eqTHN protein tagged with FLAG; red, KDEL for the endoplasmic reticulum (**A**), Rab 5 for early endosomes (**C**), Rab 7 for late endosomes (**E**) and cd63 and LAMP1 for lysosomes (**G**,**I**). Pearson correlation coefficient values for the colocalization of eqTHN and ER (**B**), early endosomes (**D**), late endosomes (**F**) and lysosomes (**H**,**J**). Error bars from three independent experiments. ***, *p* < 0.001; **, *p* < 0.01; *, *p* < 0.05; ns, not significant.

Our results show that in cells transfected with the eqTHN N-glycosylation mutants, intracellular concentrations of eqTHN proteins increased, and the amount of eqTHN at the PM decreased significantly (Figure 3A). We wanted to investigate where glycosylation mutants of eqTHN are located in cells. We analyzed the subcellular localization of WT eqTHN and its N-glycosylation mutants using the following markers: KDEL (a target peptide sequence, Lys-Asp-Glu-Leu or a closely related sequence, is present at the carboxy-terminus of soluble ER resident proteins and some membrane proteins) for the ER lumen (Figure 4A), Rab5 (Figure 4C) and Rab7 (Figure 4E) for early and late endosomes, and CD63 and LAMP1 for the lysosomes (Figure 4G,I) respectively. Quantitative analysis of the colocalization (Pearson’s coefficient calculated using the ZEN software) indicated that the N-glycosylation mutants colocalized with KDEL (Figure 4B), Rab5 (Figure 4D) and Rab7 (Figure 4F), CD63 (Figure 4H) and LAMP1 (Figure 4J), while WT eqTHN did not colocalized to these compartments. The N-glycosylation mutants also exhibited an enhanced intracellular punctate pattern and relatively higher colocalization with ER, endosomes and lysosomes compared with the WT eqTHN. These results confirm that the incompletely glycosylated eqTHN was largely retained in the ER and partially transport to lysosome, where it may then be degraded through the lysosomal pathway.

### 3.5. Anterograde Trafficking of eqTHN is Dependent on N-Glycosylation

**Figure 5:**
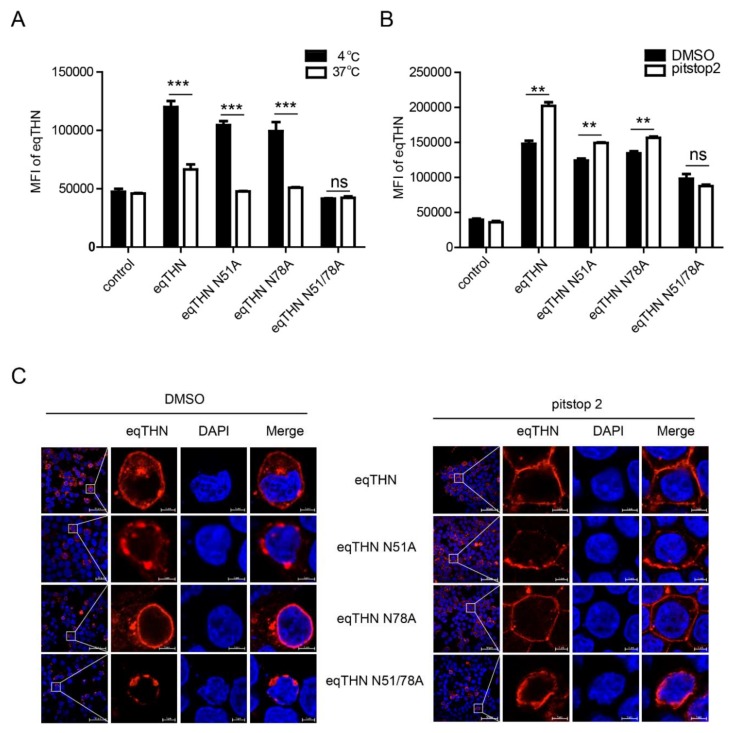
N-glycosylation modification mediates the anterograde trafficking of eqTHN in the cell. (**A**) HEK293T cells were transfected with 1μg of either pcDNA-eqTHNe-FLAG or its N-glycosylation mutants. Cells were stained with an anti-FLAG antibody and the antibody uptake was determined at 4 °C and 37 °C by flow cytometry. (**B**) HEK293T cells transfected with 1 μg of plasmids expressing either WT eqTHN or its N-glycosylation mutants, and 1 μg of plasmid expressing pcDNA3.1-FLAG as a control. At 8 h posttransfection, pitstop2 (20 μM) were added to the supernatant and incubated for 16 h and dimethyl sulfoxide (DMSO) as a control, then the cells were harvested and the mean fluorescence intensity of eqTHN on the cell surface was analyzed by flow cytometry using an anti-FLAG antibody. (**C**) HEK293T cells transfected with 1μg of plasmids expressing either WT eqTHN or its N-glycosylation mutants. At 8 h posttransfection, pitstop2 (20 μM) were added to the supernatant and incubated for 16 h and DMSO as a control, the cells were subsequently stained with an anti-FLAG antibody and the localization of eqTHN was analyzed using confocal microscopy. This experiment was performed three times, and a representative result is shown. Error bars from three independent experiments. ***, *p* < 0.001; **, *p* < 0.01; ns, not significant.

We speculated that the retention of the eqTHN N-glycosylation mutants in the cytoplasm could either be caused by an increase in the rate of tetherin endocytosis from the surface to cytoplasm or/and a block in tetherin transport from the cytoplasm to the PM. Human tetherin is recycled between the PM and the cytoplasm by the clathrin-dependent endocytosis pathway [27]. To confirm that eqTHN cycles between the PM and an intracellular pool, and to verify whether N-glycosylation impacts the efficiency of eqTHN endocytosis, we labeled cell-surface tetherin with antibody and determined the cellular localization of the protein. After incubation for 60 min at either 37 °C or at 4 °C (at which temperature endocytosis is inhibited), endocytosed antibody-labeled eqTHN and its N-glycosylation mutants were detected by FACS. The expression levels of WT eqTHN at the PM in cells incubated at 37 °C are lower than that at of those incubated at 4° C, suggesting that eqTHN is transported from the PM back to the cytoplasm through endocytosis. In addition, at 4 °C, the double N-glycosylation mutants did not localize to the PM, which suggests that the feature of mutant locating in cytoplasm is not resulted from increasing its endocytosis (Figure 5A). Because endocytosis of tetherin is clathrin-dependent [27], we were able to use pitstop2, which is an inhibitor of clatherin-dependent endocytosis, to verify whether lack of N-glycosylation can impact the efficiency of eqTHN endocytosis [28], which we checked using flow cytometry and laser confocal microscopy. The mean fluorescence intensity and expression levels of both WT eqTHN and single mutants at the PM increased significantly when treated with pitstop2 compared with the control group, while treatment with pitstop2 had no obvious effect on the double mutant (Figure 5B,C). Taken together, these results suggest that N-glycosylation probably does not impact the transport of eqTHN in the cell by affecting endocytosis, but rather by influencing the anterograde trafficking of the protein.

## 4. Discussion

Tetherin is a cellular restriction factor with broad-spectrum antiviral function that inhibits the release of viruses [14]. Current research suggests that the antiviral activity of tetherin employs a “physical tethering” model [29]. However, the detailed molecular mechanism of tetherin’s antiviral function remains unclear. In particular, the effect of N-glyosylation modifications on the antiviral activity of tetherin is still controversial. In this study, we confirmed that eqTHN has two N-glycosylation sites, and that the mutation of these two N-glycosylation sites almost completely eliminated the inhibition of virion release, possibly because of the change in subcellular localization of eqTHN.

Although the amino acid sequences of tetherin in different species are quite different, there are two N-glycosylation sites in their extracellular region that are conserved across tetherins from different animals. These two N-glycosylation sites are 27 amino acids apart, and it is suggested that N-glycosylation may be crucial for maintaining the structure, and therefore, the function of tetherin proteins. In this study, we found that mutation of N51A or N78A led to decrease of the molecular weight of eqTHN, and furthermore, that PNGase F seriously influenced the electrophoretic mobility of WT eqTHN, eqTHN N51A and eqTHN N78A, while there was no obvious impact on the eqTHN N51A/N78A mutants, indicating that eqTHN harbors two N-glycosylation (N51, N78) sites and that these are important for the electrophoretic mobility of the tetherin protein. We also demonstrated that the antiviral function of eqTHN in inhibiting EIAV virion partical release was lightly attenuated following a single mutation of an N-glycosylation site, while double N-glycosylation mutants lost nearly all their antiviral function. In addition, we found that single N-glycosylation mutants impacted the cellular localization of the tetherin, while double N-glycosylation mutants lost almost all ability to localize to the PM. Our results therefore suggest that deficiency of N-glycosylation leads to changes cellular localization in eqTHN, and that its antiviral function is weakened. Nevertheless, several researches reported that N-glycosylation of eqTHN was not required for its antiviral activity, and they did not investigate the relationship between N-glycosylation of eqTHN with its subcellular localization [20]. However, it is noteworthy that we found the double N-glycosylation mutants concentrated mainly in the cytoplasm. As we know, the antiviral mechanism of tetherin involves restricting the release of the virion by tethering it to the PM, so it is necessary for tetherin to localize to the PM for executing its full antiviral function. Hence, this study gives a conclusion that N-glycosylation is crucial for antiviral function of eqTHN by mediating its localization in cells.

Glycosylation of proteins is one of the most common post-translation modifications of proteins, in which N-glycosylation plays a critical role in protein folding, maturation and transport [30]. For instance, HMGB1 N-glycosylation is important for HMGB1 intracellular trafficking and secretion [31]. Here, we found that the cellular distribution of eqTHN and its N-glycosylation mutants were different, destruction of N-glycosylation affects eqTHN’s localization at the PM, and the mutation of N-glycosylation caused more accumulation of eqTHN in cytoplasm than WT eqTHN. Wang et al. reported a similar phenomenon that N-glycosylation mutants of corin were reduced on the surface of the transfected HEK293 cells [32]. In this study, we found that WT eqTHN showed considerable localization at the PM and moderate colocalization with the ER, which could be characteristic of newly synthesized protein, while the eqTHN mutants (single or double N-glycosylation mutants) showed punctated signals colocalization with the ER, suggesting that eqTHNs without N-glycosylation were unefficiently transported to the PM. Otherwise, eqTHN mutants colocalized with CD63, LAMP1, Rab5 and Rab7 positive compartments in cytoplasm. Because of Rab 5, Rab 7, CD63 and LAMP1 are vesicular marker which represents the typical trafficking route of endosome and lysosome [33,34,35]. It indicates that the protein caused by lacking N-glycosylation retained in the ER primarily and prevented transit through the secretory pathway to the PM and may degraded by the degradation pathway. In addition, it has been reported that Tetherin expressed at the PM will recycle to cytoplasm through endocytosis during the biological cycle of Tetherin. Hence, we compared the endocytosis efficiency between WT eqTHN and N-glycosylation mutants using classical antibody-antigen uptake assay, and the results showed that the mutation of N-glycosylation may not impacted the endocytosis efficiency. It may indicate that N-glycosylation at residues 51 and 78 are critical for eqTHN effectively anterograde transporting and the misfolded mutant’s eventual accumulation in cytoplasm results in the functional disruption of a variety of cellular processes including lysosomal degradation. In this study, we demonstrated that lack of N-glycosylation may attenuate the ability to transport to the PM while not impact on the endocytosis efficiency. In short, these observations indicated that N-glycosylation maintains effective intracellular trafficking of eqTHN to the PM.

Together, these studies suggest that the N-glycosylation of eqTHN is important for its antiviral function, and lack of N-glycan will attenuate the expression level at PM. This concept deserves further efforts to clarify whether the functional regulation of tetherin N-glycosylation was gained during evolution, as well as the potential correlation between the N-glycosylation and those viral antagonisms without the surface removal of Tetherin. Such studies may provide more insights into the molecular antiviral mechanism of tetherin.
